# Spike Mutation Profiles Associated With SARS-CoV-2 Breakthrough Infections in Delta Emerging and Predominant Time Periods in British Columbia, Canada

**DOI:** 10.3389/fpubh.2022.915363

**Published:** 2022-07-04

**Authors:** Chad D. Fibke, Yayuk Joffres, John R. Tyson, Caroline Colijn, Naveed Z. Janjua, Chris Fjell, Natalie Prystajecky, Agatha Jassem, Hind Sbihi

**Affiliations:** ^1^BC Centre for Disease Control, UBC BCCDC, Vancouver, BC, Canada; ^2^BC Center for Disease Control, Data and Analytics Services, Vancouver, BC, Canada; ^3^Public Health Laboratory, BC Center for Disease Control, Vancouver, BC, Canada; ^4^Department of Mathematics, Simon Fraser University, Burnaby, BC, Canada; ^5^School of Population and Public Health, The University of British Columbia, Vancouver, BC, Canada; ^6^Department of Pathology and Laboratory Medicine, The University of British Columbia, Vancouver, BC, Canada

**Keywords:** SARS-CoV-2, COVID-19, vaccine escape, vaccine breakthrough, spike, variants, whole genome sequencing, penalized regression

## Abstract

**Background:**

COVID-19 vaccination is a key public health measure in the pandemic response. The rapid evolution of SARS-CoV-2 variants introduce new groups of spike protein mutations. These new mutations are thought to aid in the evasion of vaccine-induced immunity and render vaccines less effective. However, not all spike mutations contribute equally to vaccine escape. Previous studies associate mutations with vaccine breakthrough infections (BTI), but information at the population level remains scarce. We aimed to identify spike mutations associated with SARS-CoV-2 vaccine BTI in a community setting during the emergence and predominance of the Delta-variant.

**Methods:**

This case-control study used both genomic, and epidemiological data from a provincial COVID-19 surveillance program. Analyses were stratified into two periods approximating the emergence and predominance of the Delta-variant, and restricted to primary SARS-CoV-2 infections from either unvaccinated individuals, or those infected ≥14 days after their second vaccination dose in a community setting. Each sample's spike mutations were concatenated into a unique spike mutation profile (SMP). Penalized logistic regression was used to identify spike mutations and SMPs associated with SARS-CoV-2 vaccine BTI in both time periods.

**Results and Discussion:**

This study reports population level relative risk estimates, between 2 and 4-folds, of spike mutation profiles associated with BTI during the emergence and predominance of the Delta-variant, which comprised 19,624 and 17,331 observations, respectively. The identified mutations cover multiple spike domains including the N-terminal domain (NTD), receptor binding domain (RBD), S1/S2 cleavage region, fusion peptide and heptad regions. Mutations in these different regions imply various mechanisms contribute to vaccine escape. Our profiling method identifies naturally occurring spike mutations associated with BTI, and can be applied to emerging SARS-CoV-2 variants with novel groups of spike mutations.

## Introduction

Severe acute respiratory syndrome coronavirus 2 (SARS-CoV-2) is the causative agent for the ongoing COVID-19 pandemic, which is responsible for 300 million infections and 5.5 million deaths worldwide (1). Public health efforts including hand washing, mask wearing, physical distancing and vaccination are associated with reductions in the spread of COVID-19 (2–5). Both Moderna mRNA-1273 and Pfizer–BioNTech BNT162b2 vaccines have shown high effectiveness at preventing severe COVID-19 related illness by around 95% (3, 5). Despite vaccination, breakthrough infections (BTI) are reported after receiving two vaccine doses. Decreased protection has been attributed to vaccine waning since becoming fully vaccinated (6) and the emergence of SARS-CoV-2 variants capable of evading neutralizing antibodies (7).

SARS-CoV-2 has diversified into many variants with sub-lineages. Several of these have been classified as Variants of Concern (VoC), which includes Alpha (B.1.1.7, Q.^*^), Beta (B.1.351), Gamma (P.1), Delta (B.1.617.2, AY.^*^) and most recently Omicron (B.1.1.529, BA.^*^) (8). These VoCs are associated with increased transmissibility, infectivity and/or breakthrough potential (9–13). Their increased fitness has been attributed, in part, to several key mutations spanning the spike protein, which is responsible for binding to the Angiotensin-converting enzyme 2 (ACE2) receptor and subsequent fusion into host cells (14). These features make the spike protein an immunodominant target for neutralizing antibodies (15). In response, spike mutations have emerged and contribute to the evasion of neutralizing antibodies and increased host-receptor affinity. For example, the D614G mutation became predominant early in the pandemic and induces a more open conformation for subsequent ACE2 binding (16). The Alpha-variant harbors several mutations including N501Y and P681R, which enhance ACE2 binding and furin cleavage, respectively (17, 18). The Beta-variant harbors these mutations with the addition of the E484K and K417N mutations, which increase the affinity to the ACE2 receptor and aid in immune escape, respectively (17, 19). Among other emerging variants, the Delta-variant has several spike mutations including T19R, G142D, E156G/Δ 157–158, L452R, T478K and D950N. The combination of these mutations support the escape of neutralizing antibodies and increased affinity to ACE2 (7, 20). With the emergence of Omicron-variant, additional novel spike mutations are still being characterized.

The majority of studies characterizing spike mutations use *in-vitro* assays, protein modeling, and convenient sampling. To date, there are limited studies which characterize vaccine escape mutations at the population level. Genome-wide association studies (GWAS) are suited for finding relationships between mutations and given phenotypes in a population. However, this approach may overlook the interaction or additive effect several mutations have on a complex phenotype. This limitation is further exacerbated by the fact that emerging SARS-CoV-2 variants introduce multiple novel mutations at a time, a phenomenon exemplified by the Omicron-variant. Therefore, we stratify isolates by spike mutation profiles (SMP) and are the first to identify spike mutations and SMPs associated with vaccine BTI within a community setting in British Columbia, Canada. Our analyses take place in two adjacent periods during the pandemic, which are the emergence and predominance of the Delta-variant in British Columbia, Canada in the context of a population with varying degrees of vaccination dosage and coverage.

## Methods

### Data Sources

We leveraged laboratory both diagnostic data, including quantitative PCR (qPCR) and whole genome sequencing (WGS), and epidemiological data from an ongoing provincial SARS-CoV-2 surveillance program previously described (21). Briefly, publicly funded diagnostic qPCR testing was widely available for symptomatic individuals and those associated with outbreaks. Testing was implemented through a network of hospital laboratories and the British Columbia Center for Disease Control (BCCDC) Public Health Laboratory (PHL), which serves as a reference laboratory. In addition, vaccination data from BC's Provincial Immunization Registry was retrieved for defining cases and controls. Testing, genomic, case and vaccination data were linked using a minimum of three key personal identifiers, including Personal Health Number (PHN), full name and date of birth. The reported prevalence of the Delta-variant in BC was used to stratify observations into Delta-variant emerging and predominant periods. The Delta emerging period (April 15th–August 31st, 2021) was defined as a period were the prevalence of this VOC increased from 0.24 to 99% of all VOCs reported in BC (22). The Delta predominant period (September 1st–November 30th, 2021) was characterized as a period with sustained prevalence around 99% of all VOCs being reported in BC (22).

### Centralized Population Level Genomic Surveillance

**Figure 1:**
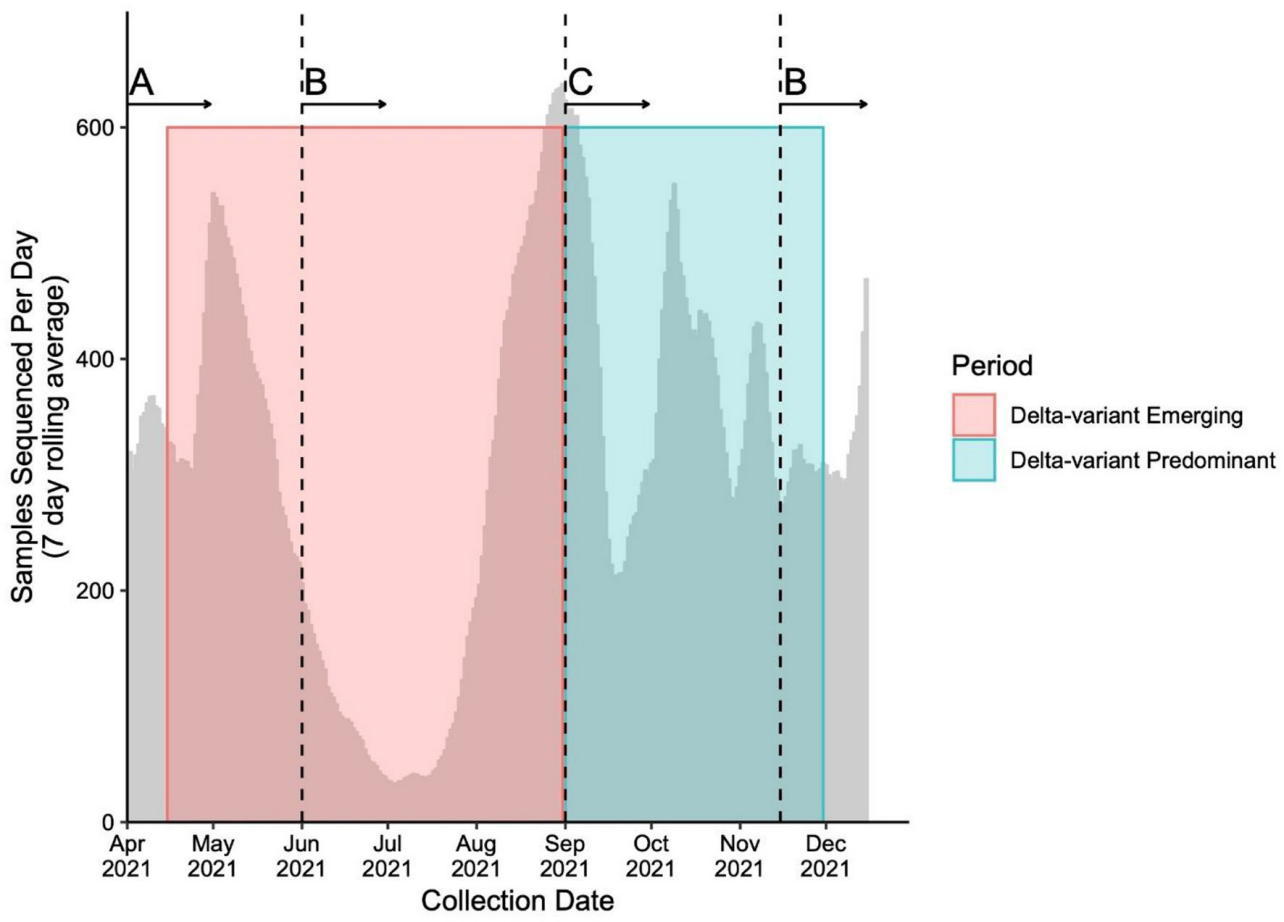
Number of samples sequenced (7 day rolling average) during study period. Sequencing strategies throughout the study periods adapted to changes in testing guidelines and sequencing capacity to capture the evolution of the pandemic. The number of averaged sequenced samples is plotted by time overlapping the Delta-variant emerging (pink box) and predominant (blue box) periods. The vertical dashed lines represent the time when whole genome sequencing (WGS) strategy changed. The sequencing strategies include **(A)** a combination of targeted qPCR-based single-nucleotide polymorphism (SNP) and WGS of between 30 and 78% of all positive SARS-CoV-2 samples, **(B)** WGS of all positive SARS-CoV-2 samples and **(C)** WGS of all positive SARS-CoV-2 samples on the first week of the month, and WGS of a representative subset of 10% of all positive SARS-CoV-2 samples for the second, third and fourth week of the month.

Throughout the study period, the genomic surveillance strategy was designed to account for the provincial testing guidelines and case load while ensuring the timely capture of emerging and circulating variants, as well as optimization of sequencing capacity. The sequencing strategy and magnitude is presented in Figure 1. Briefly, From April 15th to May 29th, 2021, a combined VOC testing strategy using both “screening” [i.e., targeted VOC single-nucleotide polymorphism (SNP) qPCR] and WGS was applied to specimens to detect and monitor VOC prevalence in British Columbia. Approximately 30–47% of all SARS-CoV-2-positive samples underwent WGS during early April, and 67–78% from April 25th to May 29th, 2021. From June 1st to August 31st, 2021, all positive SARS-CoV-2 samples in BC had WGS attempted. During period 2, all positive samples were sequenced on the first week of each month and a representative 10% of all samples were sequenced in the later weeks between September 1st and November 15th. After November 15th, all positive samples underwent WGS until the end of period 2. Samples' lineage was assigned using WGS if both SNP qPCR screening and WGS was applied. The library preparation and Illumina-based sequencing protocol use a modified version of the Freed et al. 1200bp amplicon scheme protocol, which has been previously described (21, 23, 24).

### Genomic Sequence Analysis

The BCCDC PHL used a modified ARTIC Network bioinformatics protocol and downstream analysis from the Simpson lab (https://github.com/BCCDC-PHL/ncov2019-artic-nf, https://github.com/BCCDC-PHL/ncov-tools) to process reads, align to the SARS-CoV-2 reference genome, and generate both variant calls and a consensus genome sequence (Supplementary Material). All genomic sequence information used in this study has been uploaded to GISAID under the submitter BCCDC PHL. The current study restricted analyses to non-synonymous single nucleotide polymorphisms (SNP) and insertions-deletions variants overlapping the spike region. The filtered spike variants were concatenated to construct unique spike mutation profiles (SMPs) per sample.

Population structure can confound the relationship between mutations and phenotypes of interest. Lack of adjustment can lead to the identification of lineage-defining mutations, which may not be associated with BTI. Therefore, we employed PopPUNK (25), a kmer-based genome clustering method, to generate population structure. Briefly, a PopPUNK database of genome sketches was generated using the trimmed consensus sequences from all samples collected between April 15th and August 31st. The consensus sequences were sketched using the strand-preserved and codon-phased options to account for the structure of the SARS-CoV-2 genome. Next, a lineage-based model using a nearest neighbor approach was used to cluster and assign a distinct lineage to observations by k-mer resolved genetic distance (25). These lineage assignments were used in downstream analyses to control for population structure.

### Eligibility Criteria for Defining Study Population

**Figure 2:**
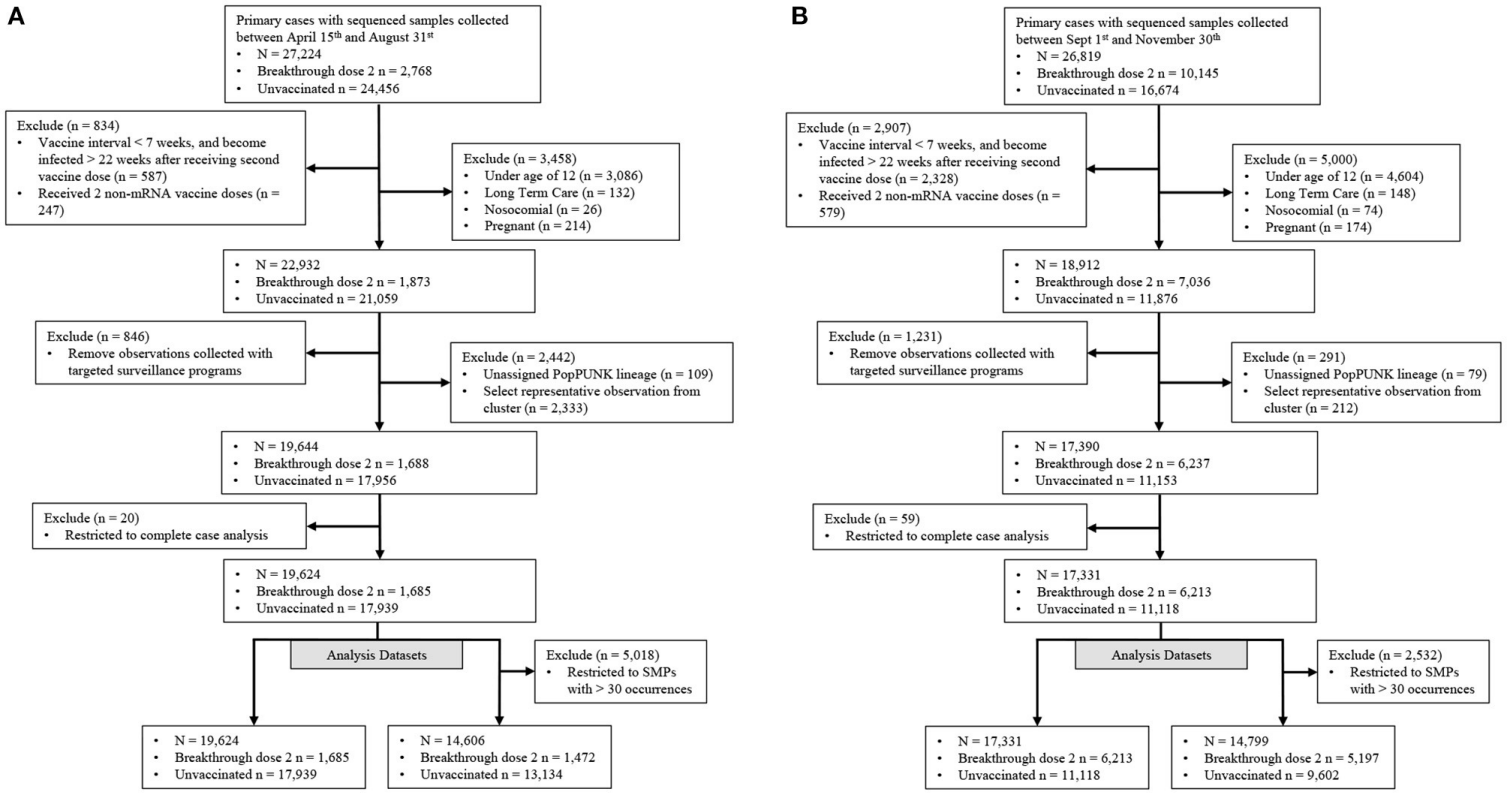
Filtration steps for samples in both Delta emerging and predominance periods. Samples were stratified into two distinct periods representing **(A)** a time when the Delta-variant was emerging and **(B)** when the Delta-variant was the major variant circulating. Targeted surveillance includes individuals regularly tested for work and travel purposes.

Our study population consisted of those with community-acquired SARS-CoV-2 primary infections with either no vaccination (controls), or those with a BTI occurring ≥14 days after receiving a second vaccine dose (cases) (Figure 2). Observations were included if collected between April 15th and November 30th, 2021 and their corresponding SARS-CoV-2 genome had <5 ambiguously called nucleotides and a breadth of coverage ≥85%. Vaccine effectiveness is associated with vaccine dosage interval, time since vaccination and vaccine type (6, 26, 27). Individuals with a BTI were kept if they met the following criteria: (i) the interval between receiving both vaccine doses was ≥ 7 weeks, (ii) became infected within 22 weeks after receiving the second dose and (iii) received at least 1 mRNA-based vaccine (Figure 2). Vaccine inclusion criteria were used to retain vaccinated individuals with sufficient protection against SARS-CoV-2. Individuals were removed if they were not eligible for vaccination in BC (ages below 12), were pregnant or known to be infected in a long-term care facility or hospital. We also removed individuals tested through targeted surveillance programs, which identified foreign temporary workers, individuals requiring regular and repeated testing, and travelers. We selected one observation from known SARS-CoV-2 clusters to limit the artificial inflation of an isolates' ability to cause breakthrough infections due to situational advantages caused by the transmission setting. The selected observation was the earliest infection of the most frequent PopPunk lineage within each cluster to approximate the strain which seeded the cluster. Finally, remaining observations were kept if they had complete sex, age, region (health authority used as proxy), collection date and genomic information recorded (Figure 2).

### Statistical Analysis

The outcome was defined as either a breakthrough or unvaccinated infection, and covariates included categorical age (12–19, 20–39, 40–59, 60–79 and ≥80), sex (male or female), region (from health authority of residence), isolate collection month, discrete lineage and unique SMP or individual spike mutations.

We employed two strategies for identifying mutations associated with BTI in both time periods. We constructed two datasets, which either consisted of an observation's mutations, or their SMP membership (Figure 2). From the mutation dataset, we removed genomic sites if the second highest allele was found in <10 cases or controls. Similarly, we removed observations in SMP with <30 occurrences and at least 3 occurring in vaccinated individuals (Figure 2). Next, elastic net penalized logistic regression was used to identify either single spike mutations, or SMPs, predictive of BTI, while adjusting for age, sex, geography, and population structure. Elastic net permits variable selection in the presence of highly correlated variables in high dimensional data. Selection is performed via coefficient shrinkage achieved by minimizing residual sum of squares and a penalty term. This penalty is a mixture of the ridge, and lasso penalties, which is the summation of either squared or absolute regression coefficients, respectively. The mixture of these penalties is controlled by a mixing parameter (α), which ranges between 0 (only applying the ridge penalty) and 1 (only applying the lasso penalty). In addition, the regularization (λ) parameter controls the contribution of the penalty term in model fitting. The caret R package (version 6.0-86) was used to perform an 80–20% train-test split of the data. The glmnet package (version 2.0–16) was used to fit a penalized model with a grid of α values between 0.3 and 1. λ parameter in each model was chosen using 10-fold cross validation to maximize the weighted area under the curve (AUC). The elastic net model was run with class weights defined as:


$$class\;weight=1-\left(\frac{\#\;in\;outcome\;class}{\#\;of\;total\;members}\right)$$

The predictive performance of each elastic net model on the test dataset was evaluated using AUC. The best performing model's non-zero penalized mutations or SMP coefficients were collected.

Lastly, we ran logistic regressions to quantify the association between identified features and breakthrough status, adjusting for age, sex, geography, and collection month. Single identified mutations were separately quantified, and their corresponding coefficient's *p*-value was corrected for multiple comparisons using the false discovery rate (FDR) adjustment. All analyses and visualizations were conducted in R, version 3.5.2 (28).

### Ethics

This study's protocol was approved by The University of British Columbia's institutional review board (REB H21-01206).

## Results

### Study Characteristics

The Delta-variant emerging and predominance period consisted of 19,624 and 17,331 individuals, respectively (Table 1). Most COVID-19 isolates were from people under 60 years old, with an even distribution between sexes (Table 1). The majority of infections in both periods occurred in unvaccinated individuals, but the frequency of BTI increased over time with the majority being associated with the Delta-variant (Table 1). The most common Delta sub-lineages for period 1 belong to AY.25 and AY.27, which constituted 69.3 and 21.2% of all Delta cases in this period, respectively. For period 2, the AY.25 and AY.27 were responsible for 72.0 and 19.8% of Delta cases.

**Table 1:** Characteristics of study population stratified by period and vaccination status.

	**Delta-variant emerging period (April 15**th**–Aug 31**st**)**			**Delta-variant predominance period (Sept 1**st**–Nov 30**th**)**		
	**Unvaccinated**	**Breakthrough dose 2**	**Overall**	**Unvaccinated**	**Breakthrough dose 2**	**Overall**
	**(*N* = 17,939)**	**(*N* = 1,685)**	**(*N* = 19,624)**	**(*N* = 11,118)**	**(*N* = 6,213)**	**(*N* = 17,331)**
**Sample type**						
Gargle	8,226 (45.9%)	791 (46.9%)	9,017 (45.9%)	5,652 (50.8%)	2,894 (46.6%)	8,546 (49.3%)
LRT^a^	5 (0.0%)	0 (0%)	5 (0.0%)	0 (0%)	1 (0.0%)	1 (0.0%)
NP^b^	9,684 (54.0%)	892 (52.9%)	10576 (53.9%)	5,444 (49.0%)	3,309 (53.3%)	8,753 (50.5%)
Other^c^	2 (0.0%)	0 (0%)	2 (0.0%)	11 (0.1%)	3 (0.0%)	14 (0.1%)
Missing	22 (0.1%)	2 (0.1%)	24 (0.1%)	11 (0.1%)	6 (0.1%)	17 (0.1%)
**Health authority**						
1	7,696 (42.9%)	465 (27.6%)	8161 (41.6%)	3,804 (34.2%)	2,420 (39.0%)	6,224 (35.9%)
2	5,668 (31.6%)	571 (33.9%)	6239 (31.8%)	3,148 (28.3%)	1,283 (20.7%)	4,431 (25.6%)
3	745 (4.2%)	63 (3.7%)	808 (4.1%)	2,161 (19.4%)	773 (12.4%)	2934 (16.9%)
4	2,861 (15.9%)	468 (27.8%)	3,329 (17.0%)	1,002 (9.0%)	1,147 (18.5%)	2,149 (12.4%)
5	969 (5.4%)	118 (7.0%)	1,087 (5.5%)	1003 (9.0%)	590 (9.5%)	1,593 (9.2%)
**Sex**						
Male	9,762 (54.4%)	788 (46.8%)	10,550 (53.8%)	5,945 (53.5%)	2,827 (45.5%)	8,772 (50.6%)
Female	8,177 (45.6%)	897 (53.2%)	9,074 (46.2%)	5,173 (46.5%)	3,386 (54.5%)	8,559 (49.4%)
**Collection month**						
2021–04	3,439 (19.2%)	0 (0%)	3,439 (17.5%)	–	–	–
2021–05	5,128 (28.6%)	1 (0.1%)	5,129 (26.1%)	–	–	–
2021–06	988 (5.5%)	5 (0.3%)	993 (5.1%)	–	–	–
2021–07	1,010 (5.6%)	64 (3.8%)	1,074 (5.5%)	–	–	–
2021–08	7,374 (41.1%)	1,615 (95.8%)	8,989 (45.8%)	–	–	–
2021–09	–	–	–	4,020 (36.2%)	1,443 (23.2%)	5,463 (31.5%)
2021–10	–	–	–	4,248 (38.2%)	2,558 (41.2%)	6,806 (39.3%)
2021–11	–	–	–	2,850 (25.6%)	2,212 (35.6%)	5,062 (29.2%)
**Age group**						
12–19	2,042 (11.4%)	46 (2.7%)	2,088 (10.6%)	1,550 (13.9%)	232 (3.7%)	1,782 (10.3%)
20–39	9,891 (55.1%)	677 (40.2%)	10,568 (53.9%)	4,470 (40.2%)	2,174 (35.0%)	6,644 (38.3%)
40–59	4,515 (25.2%)	549 (32.6%)	5,064 (25.8%)	3,392 (30.5%)	2,220 (35.7%)	5,612 (32.4%)
60–79	1,363 (7.6%)	347 (20.6%)	1,710 (8.7%)	1,504 (13.5%)	1,285 (20.7%)	2,789 (16.1%)
80+	128 (0.7%)	66 (3.9%)	194 (1.0%)	202 (1.8%)	302 (4.9%)	504 (2.9%)
**CT value**						
Mean (SD)	22.1 (4.80)	23.0 (4.92)	22.1 (4.82)	22.8 (4.64)	23.2 (4.80)	23.0 (4.70)
Median (Min, Max)	22.0 (10.0, 37.4)	22.9 (10.7, 36.4)	22.1 (10.0, 37.4)	22.9 (10.4, 36.5)	23.1 (10.4, 37.6)	23.0 (10.4, 37.6)
Missing	4,267 (23.8%)	255 (15.1%)	4,522 (23.0%)	3,312 (29.8%)	1,663 (26.8%)	4,975 (28.7%)
**Vaccine type**						
No vaccination	17,939 (100%)	0 (0%)	17,939 (91.4%)	11,118 (100%)	0 (0%)	11118 (64.2%)
Mix^d^	0 (0%)	127 (7.5%)	127 (0.6%)	0 (0%)	444 (7.1%)	444 (2.6%)
mRNA^e^	0 (0%)	1,558 (92.5%)	1,558 (7.9%)	0 (0%)	5,769 (92.9%)	5769 (33.3%)
**Lineage**						
Alpha	4,878 (27.2%)	4 (0.2%)	4,882 (24.9%)	3 (0.0%)	0 (0%)	3 (0.0%)
Beta	12 (0.1%)	0 (0%)	12 (0.1%)	0 (0%)	0 (0%)	0 (0%)
Delta	8,477 (47.3%)	1,665 (98.8%)	10,142 (51.7%)	11,111 (99.9%)	6,210 (100.0%)	17,321 (99.9%)
Gamma	3,780 (21.1%)	14 (0.8%)	3,794 (19.3%)	3 (0.0%)	1 (0.0%)	4 (0.0%)
Omicron	0 (0%)	0 (0%)	0 (0%)	0 (0%)	1 (0.0%)	1 (0.0%)
VOI^f^	288 (1.6%)	0 (0%)	288 (1.5%)	0 (0%)	0 (0%)	0 (0%)
VUM^g^	32 (0.2%)	0 (0%)	32 (0.2%)	0 (0%)	0 (0%)	0 (0%)
Other	472 (2.6%)	2 (0.1%)	474 (2.4%)	1 (0.0%)	1 (0.0%)	2 (0.0%)
Unique PopPUNK lineages	155	29	155	135	127	144

a *Lower respiratory tract sampling method*.

b *Nasopharyngeal sampling method*.

c *Other includes upper respiratory tract, nares, and other mis-specified sampling method*.

d *Receiving a combination of a mRNA vaccine (Moderna mRNA-1273 and Pfizer–BioNTech BNT162b2) and a viral-vector-based vaccine (AstraZeneca)*.

e *Receiving a combination of the Moderna mRNA-1273 and Pfizer–BioNTech BNT162b2 vaccines*.

f *Variant of interest, including the Epsilon, Eta and Mu variants*.

g *Variants under monitoring, including Iota and Kappa variants*.

### Breakthrough Infections in the Delta Emerging Period

**Figure 3:**
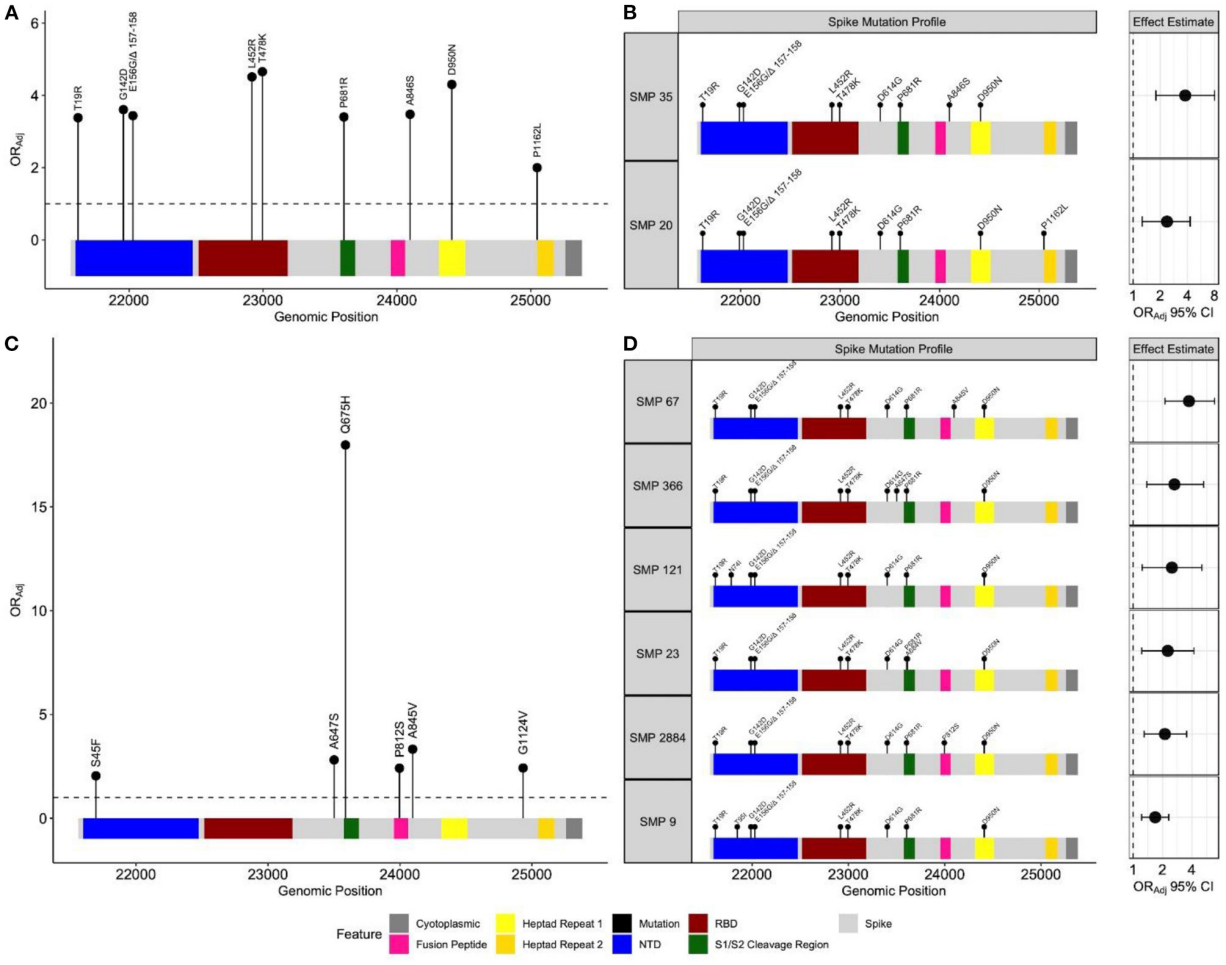
Spike mutation variants and profiles associated with breakthrough infections during the emergence and predominance of the Delta-variant. Separate elastic net models were fit to identify either individual spike variants, or spike mutation profiles (SMP) associated with breakthrough infections. **(A)** Depicts individual mutations associated with breakthrough during the emergence (*n* = 19,624) and **(C)** predominance of the Delta-variant (*n* = 17,331). The black circles represent the odds ratio for each mutation, adjusting for age, sex, health authority, and month of collection. The profiles for each SMP identified during the **(B)** emergence (*n* = 14,606) and **(D)** predominance periods (*n* = 14,799) are plotted with their corresponding odds ratio and 95% confidence intervals, adjusting for age, sex, health authority, and month of collection. The reference group SMP consisted of the most common Delta spike variants (T19R, G142D, E156G Δ 157–158, L452R, T478K, D614G, P681R and D950N). The N-terminal domain and receptor binding domain are abbreviated with NTD and RBD, respectively.

There were 729 unique positions across the spike gene harboring non-synonymous mutations. Frequency based filtration of sites resulted in the retention of 29 sites which were supplied to the penalized regression models. An elastic net model with α = 0.4 and λ = 0.0076 maximized predictive performance (AUC = 0.87). This model recognized 12 spike mutations to be most predictive of BTI (Supplementary Results). The relationship between these mutations and BTI were quantified with subsequent regression models, adjusting for age, sex, region, and collection time. All spike mutations which remained positively associated with BTI include: T19R, G142D, E156G/Δ 157-158, L452R, T478K, P681R, A846S, D950N and P1162L (Figure 3A). These identified mutations span multiple regions of the spike protein including the N-terminal domain (NTD), receptor binding domain (RBD), S1/S2 cleavage region and heptad regions (Figure 3A). Adjusted odds ratio estimates for these mutations range between 2.00 and 4.56, and are reported in Supplementary Table S1.

No isolate harbored all identified mutations positively associated with breakthrough. In response, we sought to identify naturally occurring SMPs positively associated with BTI. All SARS-CoV-2 isolates from this period were clustered into 1,218 unique SMPs. We restricted our population to individuals infected with isolates in SMP groups with frequencies ≥30 to justify multivariate analyses (total *n* = 14,606, unvaccinated *n* = 13,134, dose 2 breakthrough *n* = 1,472). This filtering resulted in further analysis of 17 SMPs for which an elastic net model with α = 0.5 and λ = 0.0085 provided the best predictive performance (AUC = 0.86). A Delta-variant SMP, containing T19R, G142D, E156G/Δ 157-158, L452R, T478K, D614G, P681R, and D950N, was used as a reference group to quantify identified SMPs association with BTI. Two SMPs were positively associated with BTI, and shared the Delta-variant mutations, with the addition of either A846S (OR_Adj_ = 2.37, 95% CI 1.26–4.29, *P*-value = 5.4e^−3^), or P1162L (OR_Adj_ = 3.78, 95% CI 1.79–8.00, *P*-value = 4.5e^−4^) amino acid mutations (Figure 3B). The frequency of these identified SMPs, stratified by periods, is reported in Supplementary Table S2.

### Breakthrough Infections in the Delta Predominance Period

For this period, the study samples contributed 732 unique positions across the spike gene, of which 50 remained after frequency pre-screening. An elastic net model, parametrized with α = 0.7 and λ = 0.0034, maximized performance (AUC = 0.67). This model identified 25 spike mutations to be predictive of BTI (Supplementary Results). The S45F, A647S, Q675H, P812S, A845V and G1124V mutations remained positively associated with BTI, and span both inter and intra functional spike regions (Figure 3C). The adjusted odds ratio for these identified mutations range between 2.04 and 18, and are reported in Supplementary Table S1. No isolate contained all identified mutations, so we employed the SMP analysis.

This time period contained 1,090 unique SMPs. Frequency filtration resulted in 36 SMPs (total *n* = 14,799, unvaccinated *n* = 9,602, dose 2 breakthrough *n* = 5,197). An elastic net model, parametrized with α = 0.3 and λ = 0.017, achieved optimal performance (AUC = 0.67), and subsequent multivariate analysis identified 6 SMPs to be positively associated with BTI, relative to a Delta-variant SMP (Figure 3D). The SMPs shared the Delta-variant mutations, with the addition of either N74I (OR_Adj_ = 2.49, 95% CI 1.24–5.10, *P*-value = 1.0e^−2^), T95I (OR_Adj_ = 1.68, 95% CI 1.22–2.31, *P*-value = 1.4e^−3^), A647S (OR_Adj_ = 2.65, 95% CI 1.38–5.26, *P*-value = 4.1e^−3^), A684V (OR_Adj_ = 2.25, 95% CI 1.22–4.19, *P*-value = 9.0e^−3^), P812S (OR_Adj_ = 2.11, 95% CI 1.30–3.53, *P*-value = 3.2e^−3^) or A845V (OR_Adj_ = 3.73, 95% CI 2.12–6.80, *P*-value = 8.9e^−6^) amino acid mutations (Figure 3D). The frequency of each identified SMP in both time periods is presented in Supplementary Table S2. The two methods showed discordant results where the individual method identified the Q675H and G1124V mutations, while the SMP approach selected the N74I, T95I, and A684V mutations.

## Discussion

Recent studies have relied on protein modeling, *in-vitro* experiments, and frequency-based trends to examine the relationship between BTI and SARS-CoV-2 spike mutations. In this work, we extend the current knowledge by examining both spike mutations, and SMPs associated with vaccine BTI in a community setting during the emergence and predominance of Delta, a SARS-CoV-2 variant of concern. Our findings corroborate Delta-defining spike mutations, along with A846S and P1162L to be associated with BTI during the emergence of the Delta-variant. We also find A647S, P812S, and A845V mutations confer additional vaccine escape potential.

The COVID-19 pandemic has seen several SARS-CoV-2 variants emerge. We found T19R, G142D, E156G/Δ 157-158, L452R, T478K, D614G, P681R, D950, A846S, and P1162L mutations to be positively associated with BTI during the emergence of the Delta-variant. These mutations cover multiple spike domains including the NTD, RBD, S1/S2 cleavage region, fusion peptide and heptad regions. This suggests various mechanisms may contribute to SARS-CoV-2 vaccine BTIs. A previous study showed full-length Delta spike protein constructs have an increased rate of membrane fusion, relative to other lineages (29). The previous study also shows T19R, G142D, and E156G/Δ 157–158 mutations decreased affinity of NTD-targeted antibodies, while L452R and T478K do not aid in the evasion of several neutralizing antibodies (29). Others suggest L452R and T478K mutations increase the stability of the ACE2-RBD complex (20). In addition to effective binding, the identified P681R mutation has been associated with increased viral fusion (18). We also identified A846S, D950N, and P1162L to be positively associated with BTIs. The A846S mutation is adjacent to the fusion peptide and both D950N and P1162L are within or proximal to heptad regions, which are important for membrane fusion (30). Both A846 and P1162 sites are associated with decreased spike protein stability (31), but their proximity to key spike domains may provide increased fitness. Interestingly, our identified SMPs harbor the exact same set of mutations. All Delta-defining spike mutations were found in these SMPs, with the addition of either the A846S, or P1162L mutations. In addition to agreeing with the individual spike analysis, the proposed SMPs analysis allows further evaluation of these novel mutations. Both methods indicate that Delta-defining spike mutations likely contributed in overcoming the previous co-dominance of the Alpha and Gamma variants in BC between April and August, 2021 (22).

In the Delta-predominant period, we identified S45F, N74I, T95I, A647S, Q675H, P812S, A845V, and G1124V to be positively associated BTIs. There is sparse information about the S45F and T95I mutations, but their position in the NTD may contribute to evasion of neutralizing antibodies. The frequency of T95I has increased overtime (32), and is present in other SARS CoV-2 variants including Omicron. The N74I has not been previously associated with breakthrough infections. This position is glycosylated and adjacent to a NTD “super site” recognized by neutralizing antibodies (33). The loss of the glycan would decrease glycan shielding, which is sparse relative to other densely glycosylated viruses (34). However, the proximity to this “super site” may be associated with increased evasion of the immune system. We also identified A647S and A845V, which are positioned between functional domains of the spike protein. These mutations increase the spike protein's stability (31, 35). Other *in-silico* studies show Q675H is associated with decrease protein stability (31), but provides increased furin affinity (36). Lastly, we identified P812S to be positively associated with BTIs, but an *in-silico* study suggests this mutation decreases spike-TMPRSS2 binding (37). This difference is likely related to reporting the isolated effect P812S has on protein interactions compared to the effect a group of mutations has on a complex phenotype. Finally, we note G1124V is positively associated with BTIs at the population level, which agrees with a previous study showing epitopes with this mutation decrease the affinity to several HLA alleles (38). This finding further highlights alternative mechanisms for immune evasion. The SMP approach reached similar conclusions in this period. However, the SMP method did not identify Q675H and G1124V, as these mutations were at low frequencies, but instead identified the T95I, N74I, and A684V mutations. The A684V is located in the S1/S2 cleavage region and could interact with the proximal P681R mutation to aid in furin binding. Interestingly, positively associated mutations did not remain associated with BTIs in both periods, except for the Delta defining mutations found with the SMP approach. This may be explained by the transient nature of mutations circulating in the population, which has shown rapid fluctuations in several spike mutations including S477N, A222V, H49Y, and V1176F (32). Furthermore, several identified mutations are characterized as destabilizing. However, protein stability has not been previously quantified in the presence of additional spike mutations, which could interact to become neutral or beneficial.

The current study has several strengths. First, we utilize both population-based epidemiological, and WGS data from prospectively collected COVID-19 samples across BC. This information allowed us to stringently define community-acquired infections, avoid misclassification bias in our outcome group, and increased external validity. Second, the sequencing strategy ensured adequate and accurate representation of circulating variants. Despite our strengths, the study has a limitation in that the majority of cases analyzed are symptomatic. This limitation may underestimate some non-synonymous spike mutations. In conclusion, we identify novel BTI mutations and propose the use of SMPs, which concur with traditional methods, prioritizes naturally occurring isolates and highlights the affect coupled mutations have on an outcome. These results extend our knowledge of SARS-CoV-2 vaccine breakthrough mutations to the population level, and provide a robust method for analyzing variants emerging with novel groups of spike mutations.

## Data Availability Statement

The datasets presented in this study can be found in online repositories. The names of the repository/repositories and accession number(s) can be found below: https://www.gisaid.org/, Submitter: BCCDC PH.

## Ethics Statement

The studies involving human participants were reviewed and approved by the University of British Columbia's institutional review board (REB H21-01206). Written informed consent from the participants' legal guardian/next of kin was not required to participate in this study in accordance with the national legislation and the institutional requirements.

## Author Contributions

HS, AJ, and NP conceptualized the study. CDF conducted the literature review, was responsible for the analyses, figure generation, and writing the manuscript. NJ, CF, and HS were responsible for acquiring demographic and epidemiological data for the population of interest. NP and JT oversaw the collection, processing, and reporting of genomic data for study subjects. CDF, YJ, and HS were responsible for data linkage, cleaning, and implementing inclusion criteria for the study. HS and YJ have reviewed the analyses. HS, AJ, NP, JT, and CC aided in the interpretation of the data. All authors reviewed and approved the final manuscript.

## Funding

The founding sources BCCDC Foundation for Public Health, Genome BC, Michael Smith Foundation for Health Research (VAC008) and Canadian Institutes for Health Research (GA1-177697) supported this study.

## Conflict of Interest

NJ participates in Abbvie Advisory Board meeting. The remaining authors declare that the research was conducted in the absence of any commercial or financial relationships that could be construed as a potential conflict of interest.

## Publisher's Note

All claims expressed in this article are solely those of the authors and do not necessarily represent those of their affiliated organizations, or those of the publisher, the editors and the reviewers. Any product that may be evaluated in this article, or claim that may be made by its manufacturer, is not guaranteed or endorsed by the publisher.
