# Supplementary material for: Spike Mutation Profiles Associated With SARS-CoV-2 Breakthrough Infections in Delta Emerging and Predominant Time Periods in British Columbia, Canada
Source: Front Public Health. 2022 Jul 4;10:915363. doi: 10.3389/fpubh.2022.915363 (PMC9289444; doi:10.3389/fpubh.2022.915363)
Supplement: Supplementary file 1 [file Data_Sheet_1.docx]

**Supplementary Material – Methods and Results**

**Supplementary Methods**

*Genomic Sequence Analysis*

The analyses used data processed using a modified ARTIC Network bioinformatics protocol (<https://github.com/BCCDC-PHL/ncov2019-artic-nf>). BWA (1) and custom scripts were used to retain reads aligning to the SARS-CoV-2 reference genome (MN908947.3). Remaining reads were further processed using Trimgalore (<https://github.com/FelixKrueger/TrimGalore>) to remove adapter sequences, low quality 3` bases and trimmed reads with a length below 20bp. BWA was used to re-align trimmed reads to the SARS-CoV-2 genome. Mapped alignments with MAPQ >=20 were down sampled to achieve a max 250X coverage if coverage was uniformly above 250X across amplicon regions. Freebayes (2) was then used to call variants and generate a consensus genome sequence with a ploidy set to 1 and the pooled-continues flag toggled. The consensus sequence was aligned to the SARS-CoV-2 genome using MAFFT (3) and 3’ and 5’ UTR sequences trimmed. Called variants were kept if the variant allele frequency was above 0.25 with >=10X coverage. Positions with multiple INDELs were processed to keep the INDEL with the highest variant allele frequency (VAF). Remaining variants were normalized with bcftools (4), and variants’ functional and amino acid consequence were annotated using SNPeff (5) against the SARS-CoV-2 genome using ncov-tools (https://github.com/BCCDC-PHL/ncov-tools). The current study restricted analyses to nonsynonymous SNP and INDEL variants overlapping the spike region. In addition, the filtered spike variants were concatenated to construct unique spike mutation profiles (SMPs) per sample.

1. Li H, Durbin R. Fast and accurate short read alignment with Burrows-Wheeler transform. Bioinformatics [Internet]. 2009 Jul 15 [cited 2022 Jan 10];25(14):1754–60. Available from: https://academic.oup.com/bioinformatics/article/25/14/1754/225615

2. Garrison E, Marth G. Haplotype-based variant detection from short-read sequencing. 2012 Jul 17 [cited 2022 Jan 10]; Available from: https://arxiv.org/abs/1207.3907

3. Katoh K, Misawa K, Kuma KI, Miyata T. MAFFT: A novel method for rapid multiple sequence alignment based on fast Fourier transform. Nucleic Acids Res [Internet]. 2002 Jul 15 [cited 2022 Jan 28];30(14):3059–66. Available from: https://pubmed.ncbi.nlm.nih.gov/12136088/

4. Danecek P, Bonfield JK, Liddle J, Marshall J, Ohan V, Pollard MO, et al. Twelve years of SAMtools and BCFtools. Gigascience [Internet]. 2021 Jan 29 [cited 2022 Jan 11];10(2):1–4. Available from: https://academic.oup.com/gigascience/article/10/2/giab008/6137722

5. Cingolani P, Platts A, Wang LL, Coon M, Nguyen T, Wang L, et al. A program for annotating and predicting the effects of single nucleotide polymorphisms, SnpEff: SNPs in the genome of Drosophila melanogaster strain w1118; iso-2; iso-3. Fly (Austin) [Internet]. 2012 [cited 2022 Jan 10];6(2):80–92. Available from: https://arxiv.org/abs/1207.3907

**Supplementary Results**

We used a multi-stage approach to identify either individual spike mutations, or spike mutation profiles (SMPs) associated with SARS-CoV-2 vaccine breakthrough infections (BTI) during periods approximating the emergence (April 15^th^ to Aug 31^st^, 2021) and predominance (Sept 1^st^ to Nov 30^th^, 2021) of the Delta-variant in British Columbia, Canada. Firstly, low frequency sites across the spike region or low frequency SMPs were removed from the analysis. Next, elastic net penalized logistic regression was used to identify single spike mutations, or SMPs predictive of breakthrough infections, adjusting for age, sex, geography and population structure. Finally, we used logistic regressions to measure the relationship between selected features and breakthrough status, adjusting for age, sex, geography and collection month. Spike mutations were quantified in separate logistic regression models, and multiple comparisons were accounted for by applying the false discovery rate adjustment to the P values.

*Delta-variant emerging Period*

The elastic net model identified L5F, T19R, G142D, E156G/∆ 157-158, R403K, L452R, T478K, P681R, A684V, A846S, D950N and P1162L mutations to be both positively associated with, and predictive of BTI. However, only the T19R, G142D, E156G/∆ 157-158, L452R, T478K, P681R, A846S, D950N and P1162L spike mutations were positively associated with BTI and remained statistically significant after FDR-correction. These remaining mutations are presented in Supplementary Table 1.

*Delta-variant Predominance Period*

The elastic net model identified Q14H, T19I, P25T, V36[LF], S45F, V70[IF], N74I, T95[SI], G181A, W2258L, P384L, G446V, E471Q, E538D, A647S, Q675H, A684V, P812S, I818V, A845V, G1124V and P1263L to be both positively associated with, and predictive of BTI. Single spike mutations remaining statistically significant after FDR-correction include: S45F, A647S, Q675H, P812S, A845V and G1124V. The summary statistics are presented in Supplementary Table 1.

**Supplementary Table 1. Individual spike mutations associated with breakthrough infections during the emergence (n = 19,624) and predominance (n = 17,331) of the Delta-variant.**

|  | **Delta-variant Emerging Period**  **(April 15^th^ to Aug 31^st^)** | | | **Delta-variant Predominance Period**  **(Sept 1^st^ to Nov 30^th^)** | | |
| --- | --- | --- | --- | --- | --- | --- |
|  | **OR_Adj_** | **95% CI** | **Q value** | **OR_Adj_** | **95% CI** | **Q value** |
| **Individual mutations** |  |  |  |  |  |  |
| T19R | 3.38 | 2.19 to 5.41 | 4.4e-7 | -- | -- | -- |
| G142D | 3.60 | 2.26 to 6.01 | 7.7e^-7^ | -- | -- | -- |
| E156G ∆ 157-158 | 3.44 | 2.17 to 5.67 | 1.1e^-6^ | -- | -- | -- |
| L452R | 4.51 | 2.72 to 7.89 | 2.1e^-7^ | -- | -- | -- |
| T478K | 4.65 | 2.81 to 8.14 | 2.0e^-7^ | -- | -- | -- |
| P681R | 3.40 | 1.91 to 6.46 | 1.1e^-4^ | -- | -- | -- |
| A846S | 3.48 | 1.67 to 7.22 | 1.6e^-3^ | -- | -- | -- |
| D950N | 4.30 | 2.59 to 7.54 | 4.2e^-7^ | -- | -- | -- |
| P1162L | 2.00 | 1.09 to 3.52 | 3.5e^-2^ | -- | -- | -- |
| S45F | -- | -- | -- | 2.04 | 1.27 to 3.30 | 3.2e^-2^ |
| A647S | -- | -- | -- | 2.81 | 1.49 to 5.51 | 2.3e^-2^ |
| Q675H | -- | -- | -- | 18.0 | 3.56 to 328 | 4.2e^-2^ |
| P812S | -- | -- | -- | 2.41 | 1.51 to 3.94 | 5.8e^-3^ |
| A845V | -- | -- | -- | 3.33 | 1.98 to 5.75 | 3.5e^-4^ |
| G1124V | -- | -- | -- | 2.42 | 1.27 to 4.71 | 5.0e^-2^ |

Adj = Odds ratio and 95% confidence intervals, adjusting for age, sex, health authority, and month of collection.

**Supplementary Table 2. Tabulation of spike mutation profiles by period*.**

|  | SMP 20 | | SMP 35 | | SMP 9 | | SMP 2884 | SMP 23 | | SMP 121 | | SMP 366 | | SMP 67 | |
| --- | --- | --- | --- | --- | --- | --- | --- | --- | --- | --- | --- | --- | --- | --- | --- |
|  | 1 | 2 | 1 | 2 | 1 | 2 | 2 | 1 | 2 | 1 | 2 | 1 | 2 | 1 | 2 |
|  | (N=62) | (N=4) | (N=33) | (N=503) | (N=210) | (N=173) | (N=76) | (N=55) | (N=44) | (N=8) | (N=35) | (N=2) | (N=42) | (N=15) | (N=61) |
| **Outcome** |  |  |  |  |  |  |  |  |  |  |  |  |  |  |  |
| Unvaccinated | 45  (72.6%) | 2  (50.0%) | 16 (48.5%) | 330  (65.6%) | 190 (90.5%) | 90  (52.0%) | 25  (32.9%) | 45 (81.8%) | 21  (47.7%) | 5 (62.5%) | 17  (48.6%) | 2  (100%) | 15  (35.7%) | 11 (73.3%) | 19  (31.1%) |
| Breakthrough dose 2 | 17  (27.4%) | 2  (50.0%) | 17 (51.5%) | 173  (34.4%) | 20  (9.5%) | 83  (48.0%) | 51  (67.1%) | 10 (18.2%) | 23  (52.3%) | 3 (37.5%) | 18  (51.4%) | 0  (0%) | 27  (64.3%) | 4  (26.7%) | 42  (68.9%) |
| **Sub-lineage** |  |  |  |  |  |  |  |  |  |  |  |  |  |  |  |
| AY.25.1 | 62 (100%) | 4  (100%) | 33 (100%) | 503 (100%) | 96 (45.7%) | 126 (72.8%) | 75 (98.7%) | 55 (100%) | 44 (100%) | 8 (100%) | 35 (100%) | 1 (50.0%) | 42 (100%) | 15 (100%) | 61 (100%) |
| AY.100 | 0 (0%) | 0 (0%) | 0 (0%) | 0 (0%) | 2 (1.0%) | 4 (2.3%) | 0 (0%) | 0 (0%) | 0 (0%) | 0 (0%) | 0 (0%) | 0 (0%) | 0 (0%) | 0 (0%) | 0 (0%) |
| AY.113 | 0 (0%) | 0 (0%) | 0 (0%) | 0 (0%) | 1 (0.5%) | 3 (1.7%) | 0 (0%) | 0 (0%) | 0 (0%) | 0 (0%) | 0 (0%) | 0 (0%) | 0 (0%) | 0 (0%) | 0 (0%) |
| AY.114 | 0 (0%) | 0 (0%) | 0 (0%) | 0 (0%) | 3 (1.4%) | 0 (0%) | 0 (0%) | 0 (0%) | 0 (0%) | 0 (0%) | 0 (0%) | 0 (0%) | 0 (0%) | 0 (0%) | 0 (0%) |
| AY.118 | 0 (0%) | 0 (0%) | 0 (0%) | 0 (0%) | 2 (1.0%) | 1 (0.6%) | 0 (0%) | 0 (0%) | 0 (0%) | 0 (0%) | 0 (0%) | 0 (0%) | 0 (0%) | 0 (0%) | 0 (0%) |
| AY.120.1 | 0 (0%) | 0 (0%) | 0 (0%) | 0 (0%) | 3 (1.4%) | 0 (0%) | 0 (0%) | 0 (0%) | 0 (0%) | 0 (0%) | 0 (0%) | 0 (0%) | 0 (0%) | 0 (0%) | 0 (0%) |
| AY.121 | 0 (0%) | 0 (0%) | 0 (0%) | 0 (0%) | 3 (1.4%) | 1 (0.6%) | 0 (0%) | 0 (0%) | 0 (0%) | 0 (0%) | 0 (0%) | 0 (0%) | 0 (0%) | 0 (0%) | 0 (0%) |
| AY.4 | 0 (0%) | 0 (0%) | 0 (0%) | 0 (0%) | 2 (1.0%) | 12 (6.9%) | 0 (0%) | 0 (0%) | 0 (0%) | 0 (0%) | 0 (0%) | 0 (0%) | 0 (0%) | 0 (0%) | 0 (0%) |
| B.1.617.2 | 0 (0%) | 0 (0%) | 0 (0%) | 0 (0%) | 98 (46.7%) | 10 (5.8%) | 0 (0%) | 0 (0%) | 0 (0%) | 0 (0%) | 0 (0%) | 0 (0%) | 0 (0%) | 0 (0%) | 0 (0%) |
| AY.103 | 0 (0%) | 0 (0%) | 0 (0%) | 0 (0%) | 0 (0%) | 1 (0.6%) | 0 (0%) | 0 (0%) | 0 (0%) | 0 (0%) | 0 (0%) | 0 (0%) | 0 (0%) | 0 (0%) | 0 (0%) |
| AY.109 | 0 (0%) | 0 (0%) | 0 (0%) | 0 (0%) | 0 (0%) | 3 (1.7%) | 0 (0%) | 0 (0%) | 0 (0%) | 0 (0%) | 0 (0%) | 0 (0%) | 0 (0%) | 0 (0%) | 0 (0%) |
| AY.111 | 0 (0%) | 0 (0%) | 0 (0%) | 0 (0%) | 0 (0%) | 1 (0.6%) | 0 (0%) | 0 (0%) | 0 (0%) | 0 (0%) | 0 (0%) | 0 (0%) | 0 (0%) | 0 (0%) | 0 (0%) |
| AY.117 | 0 (0%) | 0 (0%) | 0 (0%) | 0 (0%) | 0 (0%) | 6 (3.5%) | 0 (0%) | 0 (0%) | 0 (0%) | 0 (0%) | 0 (0%) | 0 (0%) | 0 (0%) | 0 (0%) | 0 (0%) |
| AY.39 | 0 (0%) | 0 (0%) | 0 (0%) | 0 (0%) | 0 (0%) | 1 (0.6%) | 0 (0%) | 0 (0%) | 0 (0%) | 0 (0%) | 0 (0%) | 0 (0%) | 0 (0%) | 0 (0%) | 0 (0%) |
| AY.39.1 | 0 (0%) | 0 (0%) | 0 (0%) | 0 (0%) | 0 (0%) | 2 (1.2%) | 0 (0%) | 0 (0%) | 0 (0%) | 0 (0%) | 0 (0%) | 0 (0%) | 0 (0%) | 0 (0%) | 0 (0%) |
| AY.42 | 0 (0%) | 0 (0%) | 0 (0%) | 0 (0%) | 0 (0%) | 2 (1.2%) | 0 (0%) | 0 (0%) | 0 (0%) | 0 (0%) | 0 (0%) | 0 (0%) | 0 (0%) | 0 (0%) | 0 (0%) |
| AY.44 | 0 (0%) | 0 (0%) | 0 (0%) | 0 (0%) | 0 (0%) | 0 (0%) | 1 (1.3%) | 0 (0%) | 0 (0%) | 0 (0%) | 0 (0%) | 1 (50.0%) | 0 (0%) | 0 (0%) | 0 (0%) |

*1: Delta-variant Emergence period; 2: Delta-variant Predominance period
